# Plexus Slim®-Induced Immune Thrombocytopenic Purpura

**DOI:** 10.7759/cureus.11413

**Published:** 2020-11-10

**Authors:** Chandler Graf, Mohamed Elmassry, Victoria M Chu, Dushyant Pawar, Lukman Tijani

**Affiliations:** 1 Internal Medicine, Texas Tech University Health Sciences Center, Lubbock, USA; 2 Hematology and Oncology, Texas Tech University Health Sciences Center, Lubbock, USA

**Keywords:** itp, drug-induced itp, supplement

## Abstract

Primary immune thrombocytopenic purpura (ITP) is a common cause of thrombocytopenia. Due to the many possible precipitating factors, the diagnostic approach can be complex in nature. Much of the published literature on drug-induced ITP (DITP) report on quinine-induced thrombocytopenia. Here we present a case of the proposed dietary cause of DITP by the weight loss supplement Plexus® which contains two potential thrombocytopenia-causing compounds, garcinia cambogia fruit extract, and chromium polynicotinate. This case highlights how a thorough patient history, including evaluation of supplement use and dietary habits, can be of the utmost importance in the workup of ITP.

## Introduction

Primary immune thrombocytopenic purpura (ITP) is an autoimmune disease caused by autoantibodies directed against platelet antigens, characterized by thrombocytopenia and increased risk of bleeding. Although the pathogenesis of the disease is not fully understood, it has been proposed that the pathogenic production of autoantibodies to the patient’s platelet membrane glycoproteins, specifically GPIIb/IIIa, is the primary pathologic feature of the disease [1,2]. The subsequent splenic destruction of autoantibody tagged platelets is accordingly the cause of the thrombocytopenia. Additionally, multiple mechanisms have been proposed for secondary ITP, defined by the destruction of platelets in the absence of primary autoimmune autoantibody production [3]. With studies estimating the incidence of the disease to be approximately eight per 100,000 in the United States, ITP is a relevant disease process that is routinely discovered and discussed in the hospital setting [4].

Of specific interest to this report are the documented cases of secondary ITP induced by dietary compounds, categorized as drug-induced ITP (DITP). While being a relatively rare cause of ITP, dietary consumption of certain compounds is a well-reported precipitating event [5,6]. Most notable are the reports of quinine-induced thrombocytopenia, a compound derived from the Cinchona trees of Peru, used for the treatment of malaria and babesiosis [7]. Incidentally, it is also available in tonic water, a liquid drink commonly consumed.

Here we report a related instance of a proposed dietary cause of DITP caused by the use of a common weight loss supplement, Plexus®. This supplement is a non-FDA approved drug marketed for weight loss and appetite suppression. Plexus contains both garcinia cambogia fruit extract, and chromium polynicotinate, both compounds hypothesized to be a potential cause of thrombocytopenia [8].

## Case presentation

A 30-year-old female, with a past medical history of polycystic ovarian syndrome and hypertension, presented to an internal medicine clinic for a chief complaint of “easy bruising” for approximately three weeks in duration. After complete blood count (CBC) and comprehensive metabolic panel (CMP) were completed, the patient was subsequently found to have a platelet count of eight-thousand and mild transaminitis with alanine aminotransferase (ALT)/ aspartate aminotransferase (AST) of 99 and 63 respectively. She was immediately sent for hospital evaluation and admission. The patient endorsed mild bleeding of the gums after brushing her teeth over the past three weeks as well as two episodes of epistaxis during this same time period. The patient denied any pertinent family and surgical history. Additionally, she stated that she had never had easy bruising or a confirmed episode of thrombocytopenia before. The patient’s medications were Apri® (desogestrel and ethynyl estradiol) and Metformin daily. On questioning dietary habits and history the patient reported that six-eight weeks prior to this presentation she was taking Plexus-SLIM® as a weight loss supplement. The patient's review of systems was otherwise negative and she stated that she felt like she was in excellent health other than this current presentation of thrombocytopenia. On physical exam, vital signs were all within normal limits, and no abnormal findings were observed other than a petechial rash extending from the patient’s abdomen to the legs bilaterally. 

A chest X-Ray and abdominal ultrasound were done during hospital stay which revealed moderate cardiomegaly, and cholelithiasis without gallbladder thickening respectively. Additionally, an abdominal ultrasound revealed that the spleen was of normal size. A peripheral blood smear done during admission confirmed thrombocytopenia and revealed the presence of giant platelets, suggesting a mechanism of either peripheral platelet destruction or splenic sequestration. No schistocytes present on peripheral smear. The patient was tested for HIV, hepatitis B, and helicobacter pylori, all of which were negative. Additionally, a rheumatoid factor and antinuclear antibody (ANA) were ordered to test for the presence of autoimmune disease. These tests returned negative.

With consideration of laboratory, radiologic findings, as well as physical exam the diagnosis of ITP was made. The patient was subsequently treated with Prednisone 100mg 1x daily and IVIG 1g/kg for two days. Over the course of three days, the patient’s platelets increased to 53 thousand. The patient was discharged on current prednisone dose with plans to gradually taper as well as follow up appointments in the clinic setting.

## Discussion

The role of precipitating factors and inciting events has become an important aspect of the treatment and prevention of ITP. It has been observed and well reported that by means of molecular mimicry specific viral infections may play an important role as the inciting event of secondary ITP. Bacterial infections, such as helicobacter pylori, have also been shown to be a potential cause in a similar way to viral infections [9-11]. It is suspected that when considering a bacterial infection as the cause of secondary ITP the lipopolysaccharide (LPS) found on the bacterial cell may play a direct role in the pathogenic human response [12]. Other possible precipitating factors include immune altering diseases such as antiphospholipid syndrome, systemic lupus erythematous, common variable immunodeficiency, and rheumatoid arthritis. While the mechanism of these immunologic diseases role in ITP is not completely understood it is proposed that T-cell mediated cytotoxicity, and/or regulatory T-cells are responsible [13].

While the potential causes of secondary ITP discussed above are the most common, they are by no means an exhaustive list and many other causes of ITP are reported in the current literature. Of these many reported causes of ITP, a small percentage are due to certain drugs and chemical compounds found in dietary supplements. Although multiple compounds have been reported we discuss chromium polynicotinate as well as garcinia cambogia extract as a possible cause for this patient’s presentation [14-17].

Chromium polynicotinate has been thoroughly investigated for its potential to encourage weight loss and decrease appetite. Although, randomized trials have failed to prove its efficacy it is still ubiquitously used in many of the top-selling weight-loss diets and supplements [15]. A case report put forth by Cerulli et al. discusses the potential pathogenic side effects of this supplemental drug. These side effects observed included renal failure, thrombocytopenia, liver toxicity, and hemolytic anemia [16,18]. Of special consideration to this specific case, is the fact that in the case presented by Cerulli et al. the thrombocytopenia was not observed until months after the initial ingestion. A similar timetable is seen in the patient reported here.

Garcinia cambogia is a fruit native to India and southwest Asia and contains the chemical compound hydroxycitric acid (HCA) which has been studied for its effects on weight loss appetite suppression [19]. Accordingly, it has been used and become widely available in products marketed to help consumers lose weight, such as Hydroxycut® and Plexus®. Both the fruit extract itself as well as HCA have been postulated as a potential cause of hepatotoxicity when consumed in sufficient amounts. This serves as a potential cause of the elevated liver enzymes observed in this patient. Although sufficient studies have not been done to evaluate a connection between garcinia cambogia and DITP there is adequate information to suggest that this chemical compound may play a role in thrombocytopenia [8,14,20].

While it is a less common cause of thrombocytopenia, DITP represents an important subsection of ITP pathology that should be considered given the appropriate clinical scenario. Numerous reports are available in the literature suggesting a possible link between chemical compounds available in weight loss supplements such as Plexus® and DITP. While no definitive link has been made the need for further studies and observation is imperative. The importance of clinical suspicion in addition to a thorough patient history cannot be overstated and is crucial to the diagnosis of DITP.

## Conclusions

DITP is often a challenging diagnosis to make in the context of thrombocytopenia. While the pathology of ITP is often considered, deciding on a precipitating event is difficult and complex in nature. A thorough and complete history of the patient, including dietary habits, is a crucial diagnostic tool in the setting of thrombocytopenia. Additionally, increasing patient awareness of the potential dangers of non-FDA regulated supplements is an important aspect of patient education and overall patient care.

## References

[1] Cines DB, Blanchette VS (2002). Immune thrombocytopenic purpura. N Engl J Med.

[2] Rodeghiero F, Stasi R, Gernsheimer T (2009). Standardization of terminology, definitions and outcome criteria in immune thrombocytopenic purpura of adults and children: report from an international working group. Blood.

[3] Kuwana M, Okazaki Y, Ikeda Y (2009). Splenic macrophages maintain the anti-platelet autoimmune response via uptake of opsonized platelets in patients with immune thrombocytopenic purpura. J Thromb Haemost.

[4] Terrell DR, Beebe LA, Neas BR (2012). Prevalence of primary immune thrombocytopenia in Oklahoma. Am J Hematol.

[5] Aster RH, Curtis BR, McFarland JG, Bougie DW (2009). Drug-induced immune thrombocytopenia: pathogenesis, diagnosis, and management. J Thromb Haemost.

[6] Aster RH, Bougie DW (2007). Drug-induced immune thrombocytopenia. N Engl J Med.

[7] Bougie DW, Wilker PR, Aster RH (2006). Patients with quinine-induced immune thrombocytopenia have both "drug-dependent" and "drug-specific" antibodies. Blood.

[8] Sikka G, Gupta A, Bachan M, Khan Z (2016). "Uber-Trim" causes immune thrombocytopenic purpura: a life threatening case report. Chest.

[9] Wright JF, Blanchette VS, Wang H (1996). Characterization of platelet-reactive antibodies in children with varicella-associated acute immune thrombocytopenic purpura (ITP). Br J Haematol.

[10] Zhang W, Nardi MA, Borkowsky W, Zongdong L, Karpatkin S (2009). Role of molecular mimicry of hepatitis C virus protein with platelet GPIIIa in hepatitis C-related immunologic thrombocytopenia. Blood.

[11] Stasi R, Provan D (2008). Helicobacter pylori and chronic ITP. Hematology Am Soc Hematol Educ Program.

[12] Semple JW, Aslam R, Kim M, Speck ER, Freedman J (2007). Platelet-bound lipopolysaccharide enhances Fc receptor-mediated phagocytosis of IgG-opsonized platelets. Blood.

[13] Mahévas M, Chiche L, Uzunhan Y (2011). Association of sarcoidosis and immune thrombocytopenia: presentation and outcome in a series of 20 patients. Medicine (Baltimore).

[14] Dara L, Hewett J, Lim JK (2008). Hydroxycut hepatotoxicity: a case series and review of liver toxicity from herbal weight loss supplements. World J Gastroenterol.

[15] Tian H, Guo X, Wang X, He Z, Sun R, Ge S, Zhang Z (2013). Chromium picolinate supplementation for overweight or obese adults. Cochrane Database Syst Rev.

[16] Cerulli J, Grabe DW, Gauthier I, Malone M, McGoldrick MD (1998). Chromium picolinate toxicity. Ann Pharmacother.

[17] George JN (2009). Drug-induced thrombocytopenia: pathogenesis, evaluation, and management. Hematology.

[18] Royer DJ, George JN, Terrell DR (2010). Thrombocytopenia as an adverse effect of complementary and alternative medicines, herbal remedies, nutritional supplements, foods, and beverages. Eur J Haematol.

[19] Haber SL, Awwad O, Phillips A, Park AE, Pham TM (2018). Garcinia cambogia for weight loss. Am J Health Syst Pharm.

[20] Melendez-Rosado J, Snipelisky D, Matcha G, Stancampiano F (2015). Acute hepatitis induced by pure garcinia cambogia. J Clin Gastroenterol.

